# Synthesis, crystal structure and Hirshfeld surface analysis of tetra­aqua­bis­(isonicotinamide-κ*N* ^1^)cobalt(II) fumarate

**DOI:** 10.1107/S205698901800107X

**Published:** 2018-01-26

**Authors:** Sevgi Kansiz, Zainab M Almarhoon, Necmi Dege

**Affiliations:** a Ondokuz Mayıs University, Faculty of Arts and Sciences, Department of Physics, 55139, Samsun, Turkey; b King Saud University, Faculty of Science, Department of Chemistry, Riyadh, Saudi Arabia

**Keywords:** crystal structure, fumaric acid, isonicotinamide, cobalt(II), Hirshfeld surfaces

## Abstract

The reaction of cobalt(II) with fumaric acid (H_2_fum) and isonicotinamide in a basic solution produces the title salt, [Co(C_6_H_6_N_2_O)_2_(H_2_O)_4_](C_4_H_2_O_4_). In the complex cation, the Co^II^ atom, located on an inversion centre, is coordinated by two isonicotinamide and four water mol­ecules in a distorted N_2_O_4_ octa­hedral geometry. The fumarate anion is located on another inversion centre and is linked to neighbouring complex cations *via* O—H⋯O and N—H⋯O hydrogen bonds and weak C—H⋯O hydrogen bonds. In the crystal, the complex cations are further linked by O—H⋯O, N—H⋯O and weak C—H⋯O hydrogen bonds, forming a three-dimensional supra­molecular architectecture. Hirshfeld surface analyses (*d*_norm_ surfaces and two-dimensional fingerprint plots) for the title compound are presented and discussed.

## Abstract 2

In the complex cation, the Co^II^ atom, located on an inverse centre, is coordinated by two isonicotinamide and four water mol­ecules in a distorted O_4_N_2_ octa­hedral geometry. The fumarate anion is located on another inverse centre and is linked to neighbouring complex cations *via* O—H⋯O and O—H⋯N hydrogen bonds and weak C—H⋯O hydrogen bonds. In the crystal, the complex cations are further linked by O—H⋯O, N—H⋯O an weak C—H⋯O hydrogen bonds, forming a three-dimensional supra­molecular architectecture.

## Chemical context   

Metal carboxyl­ates have attracted intense attention because of their inter­esting framework topologies (Rao *et al.*, 2004 ▸). Among metal carboxyl­ates, fumarate dianions (fum) have good conformational freedom and they possess some desirable features such as being versatile ligands because of the four electron-donor oxygen atoms they carry, and their ability to link inorganic moieties (Zheng *et al.*, 2003 ▸). Moreover, metal fumarates exhibit interesting structural varieties.
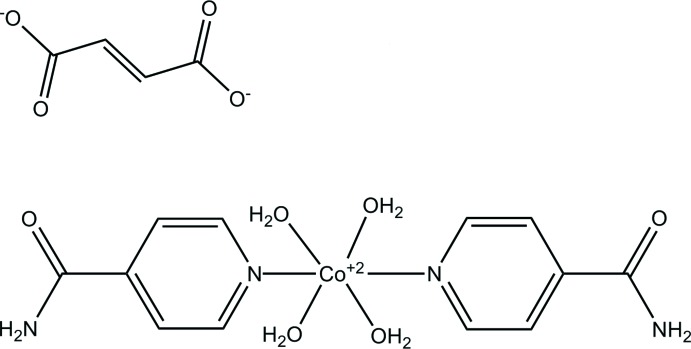


Di­carb­oxy­lic acids such as fumaric acid and amides have been particularly useful in creating many supra­molecular structures involving isonicotinamide and a variety of carb­oxy­lic acid mol­ecules (Vishweshwar *et al.*, 2003 ▸; Aakeröy *et al.*, 2002 ▸). Di­carb­oxy­lic acid ligands are utilized in the synthesis of a wide variety of metal carboxyl­ates. For this reason they have been investigated extensively, both experimentally and computationally. We describe herein the synthesis, structural features and Hirshfeld surface analysis of the title salt.

## Structural commentary   

**Figure 1:**
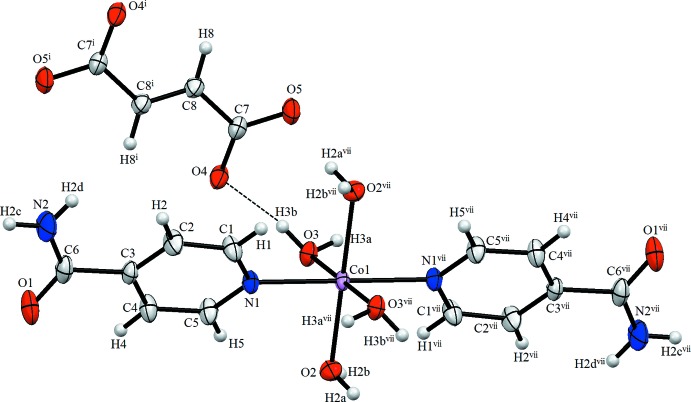
The mol­ecular structure of the title compound, showing the atom labelling. Displacement ellipsoids are drawn at the 50% probability level. [Symmetry codes: (i) –*x* + 1, −*y* + 1, −*z* + 1; (vii) –*x* + 1, −*y* + 1, −*z* + 2.]

The mol­ecular structure of the title compound is illustrated in Fig. 1 ▸. The Co^II^ cation and midpoint of the C=C bond of the fumarate anion are each located on an inversion centre. In the complex cation, the Co^II^ atom is coordinated to two isonicotinamide ligands and four water mol­ecules in a distorted N_2_O_4_ octa­hedral geometry. The fumarate anion interacts with neighboring complex cations *via* O—H⋯O and N—H⋯O hydrogen bonds and weak C—H⋯O hydrogen bonds (Table 1 ▸).

## Supra­molecular features   

**Figure 2:**
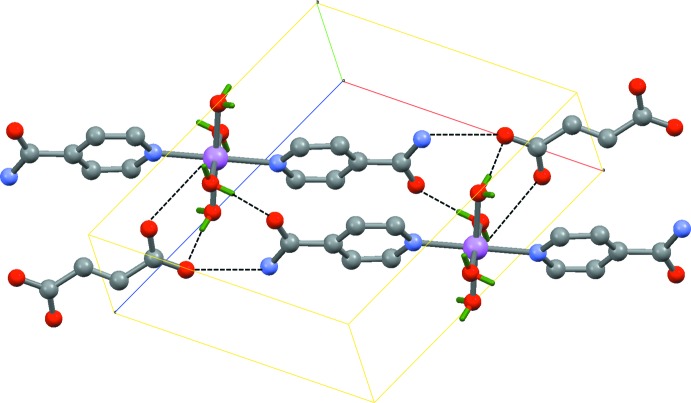
Packing of the title compound in the unit cell. Dashed lines indicate hydrogen bonds.

In the crystal, the fumarate anions and complex cations are linked by O—H⋯O, N—H⋯O and C—H⋯O hydrogen bonds; the complex cations also interact with each other through O—H⋯O, N—H⋯O and C—H⋯O hydrogen bonds, forming a three-dimensional supra­molecular architecture (Table 1 ▸, Fig. 2 ▸).

## Hirshfeld surface analysis   

**Figure 3:**
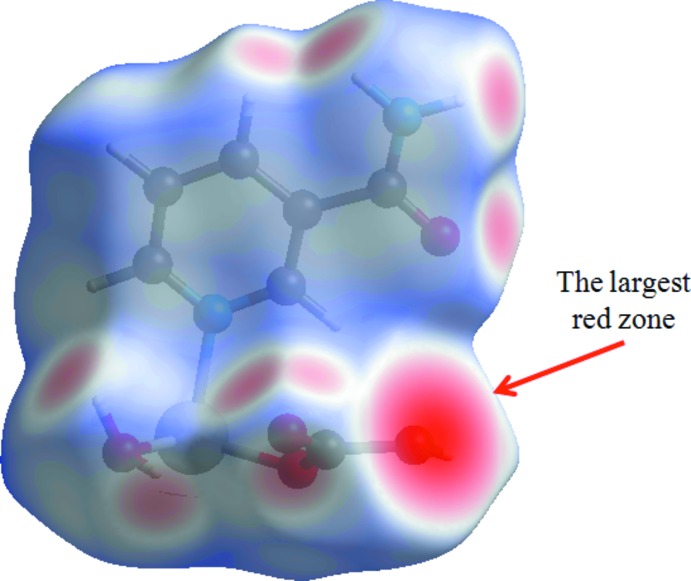
The Hirshfeld surface of the title compound mapped with *d*_norm_. The red spots indicate the regions of the donor–acceptor inter­actions.

*Crystal Explorer 17.5* (Turner *et al.*, 2017 ▸) was used to analyse the inter­actions in the crystal and fingerprint plots mapped over *d*_norm_ (Figs. 3 ▸ and 4 ▸) were generated. The contact distances to the closest atom inside (*d_i_*) and outside (*d_e_*) of the Hirshfeld surface are used to analyse the inter­molecular inter­actions *via* the mapping of *d*_norm_. The mol­ecular Hirshfeld surfaces were obtained using a standard (high) surface resolution with the three-dimensional *d*_norm_ surfaces mapped over a fixed colour scale of −1.227 (red) to 1.279 (blue). Many studies on Hirshfeld surfaces can be found in the literature (see, for example, Şen *et al.*, 2018 ▸; Yaman *et al.*, 2018 ▸).

In a *d*_norm_ surface, any inter­molecular inter­actions will appear as red spots. The red spots indicate the regions of donor–acceptor inter­actions. There are many red spots in the *d*_norm_ surface (Fig. 3 ▸), which are usually on the O-acceptor atoms involved in the inter­actions listed in Table 1 ▸. Strong hydrogen-bond inter­actions, such as O—H⋯O, are seen as a bright-red areas on the Hirshfeld surfaces (Şen *et al.*, 2017 ▸).

**Figure 5:**
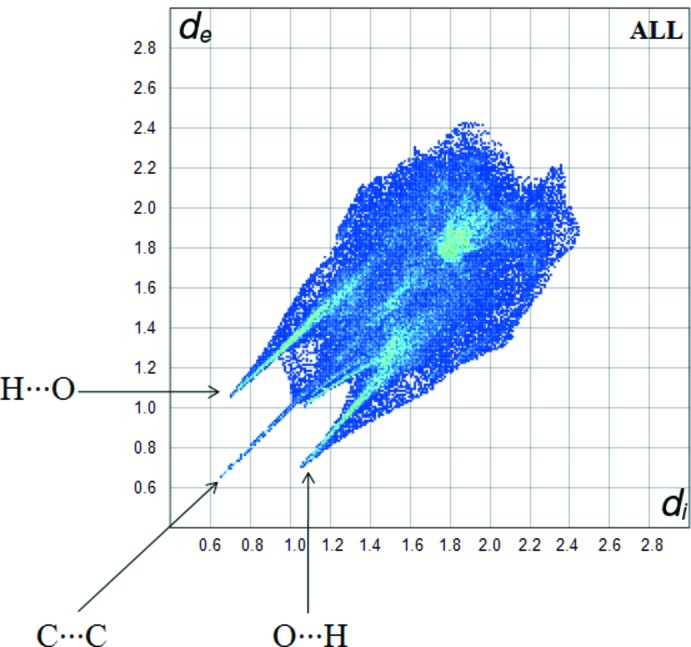
A fingerprint plot of the title complex.

**Figure 6:**
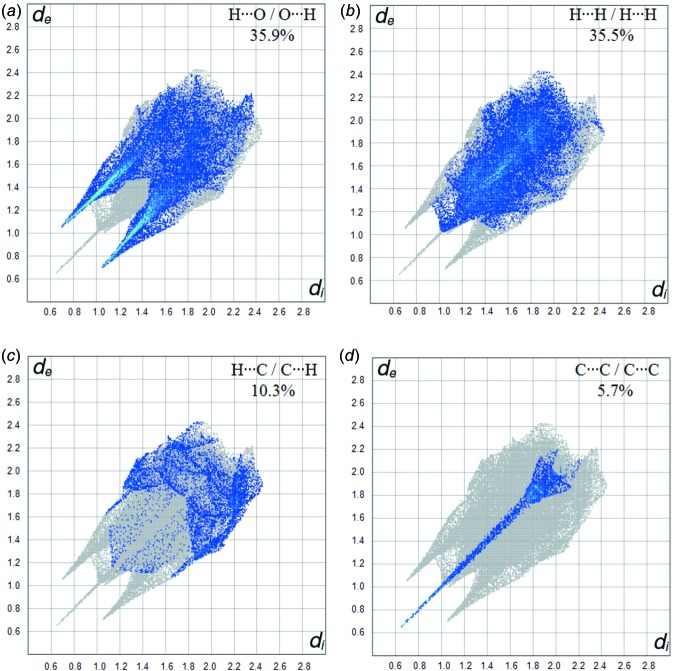
(*a*) O⋯H/H⋯O, (*b*) H⋯H/H⋯H, (*c*) C⋯H/H⋯C and (*d*) C⋯C/C⋯C contacts in the title complex, showing the percentages of contacts contributing to the total Hirshfeld surface area.

The fingerprint plot for the title complex is presented in Fig. 5 ▸. The H⋯H inter­actions appear in the middle of the scattered points in the two-dimensional fingerprint plots with an overall contribution to the Hirshfeld surface of 35.5% (Fig. 6 ▸ *b*). The contribution from the O⋯H/H⋯O contacts, corresponding to C—H⋯O, N—H⋯O and O—H⋯O inter­actions, is represented by a pair of sharp spikes characteristic of a strong hydrogen-bond inter­action (35.9%) (Fig. 6 ▸ *a*). The C⋯C/C⋯C contacts have a sharp spike between the O⋯H and H⋯O spikes (5.7%) (Fig. 6 ▸ *d*). The contribution of the other inter­molecular contacts to the Hirshfeld surfaces is C⋯H/H⋯C (10.3%) (Fig. 6 ▸ *c*).

## Database survey   

A search of the Cambridge Structural Database for fumaric acid and isonicotinamide revealed the presence of seven structures: isonicotinohyrazide nicotinamide fumaric acid (Aitipamula *et al.*, 2013 ▸), *catena*-poly[[aqua­bis­[*N*-(pyridin-3-yl)isonicotinamide-κ*N* ^1^)copper(II)]-(μ_2_-fumarato-κ*O*,*O*′)-(Qiblawi & LaDuca, 2012 ▸), bis­(isonicotinamide) fumaric acid (Aakeröy *et al.*, 2002 ▸), *catena*-[bis­(μ_2_-fumarato)bis­(μ_2_-3-pyridyl­isonicotinamide)­dizinctrihydrate] (Uebler *et al.*, 2013 ▸) and *catena*-[bis­(μ-but-2-enedioato)bis­(μ-pyridine-4-carbohydrazide)dizinc(II)] (Naskar *et al.*, 2017 ▸). In these compounds, the C—H⋯O hydrogen bonds have H⋯O distances ranging from 2.56 to 3.59 Å and C⋯O distances ranging from 3.27 to 3.96 Å. The N—H⋯O hydrogen bonds have H⋯O distances ranging from 1.86 to 2.33 Å and N⋯O distances ranging from 2.82 to 3.15 Å.

## Synthesis and crystallization   

An aqueous solution of fumaric acid (26 mmol, 3 g) in water was added to a solution of NaOH (52 mmol, 2.07 g) while stirring. A solution of CoCl_2_·6H_2_O (25 mmol, 6.19 g) in water was added. The reaction mixture was stirred for an hour at room temperature. The pink mixture was filtered and left to dry. The pink crystals (0.88 mmol, 0.20 g) were dissolved in water and added to an aqueous solution of isonicotinamide (1.75 mmol, 0.21 g). The resulting suspension was filtered and allowed to crystallize for five weeks at room temperature yielding orange block-shaped crystals suitable for X-ray diffraction analysis.

## Refinement   

Crystal data, data collection and structure refinement details are summarized in Table 2 ▸. The N-bound and C-bound hydrogen atoms were positioned geometrically and treated as riding: N—H = 0.86 Å and C—H = 0.93 Å with *U*_iso_(H) = 1.2*U*_eq_(C,N). Water H atoms were found in a difference-Fourier map, restrained with O—H = 0.85 Å and refined with *U*_iso_(H) = 1.5*U*_eq_(O).

## Supplementary Material

Crystal structure: contains datablock(s) I, global. DOI: 10.1107/S205698901800107X/xu5915sup1.cif

Structure factors: contains datablock(s) I. DOI: 10.1107/S205698901800107X/xu5915Isup2.hkl

CCDC reference: 1561543

Additional supporting information:  crystallographic information; 3D view; checkCIF report

## Figures and Tables

**Figure 4:**
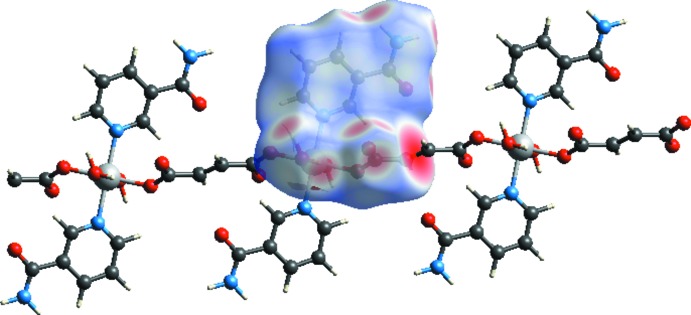
*d*_norm_ mapped on the Hirshfeld surfaces for the title structure.

**Table 1:** Hydrogen-bond geometry (Å, °)

*D*—H⋯*A*	*D*—H	H⋯*A*	*D*⋯*A*	*D*—H⋯*A*
O2—H2*A*⋯O5^i^	0.86	1.96	2.814 (2)	171
O2—H2*B*⋯O4^ii^	0.86	1.88	2.7165 (19)	165
O3—H3*A*⋯O1^iii^	0.86	1.95	2.792 (2)	168
O3—H3*B*⋯O4	0.86	1.82	2.6652 (19)	172
N2—H2*C*⋯O5^iv^	0.86	2.13	2.955 (2)	160
N2—H2*D*⋯O1^v^	0.86	2.47	3.288 (3)	159
C1—H1⋯O4^vi^	0.93	2.41	3.322 (2)	168
C2—H2⋯O1^v^	0.93	2.30	3.225 (3)	173

Symmetry codes: (i) 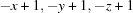; (ii) 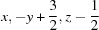; (iii) 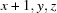; (iv) 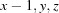; (v) 
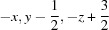
; (vi) 
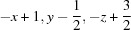
.

**Table 2:** Experimental details

Crystal data	
Chemical formula	[Co(C_6_H_6_N_2_O)_2_(H_2_O)_4_](C_4_H_2_O_4_)
*M*_r_	489.30
Crystal system, space group	Monoclinic, *P*2_1_/*c*
Temperature (K)	296
*a*, *b*, *c* (Å)	9.6914 (10), 10.0106 (11), 11.3811 (12)
β (°)	113.416 (3)
*V* (Å^3^)	1013.22 (19)
*Z*	2
Radiation type	Mo *K*α
μ (mm^−1^)	0.91
Crystal size (mm)	0.25 × 0.19 × 0.16

Data collection	
Diffractometer	Bruker APEXII CCD
Absorption correction	Analytical (*X-RED32*; Stoe & Cie, 2002 ▸)
*T*_min_, *T*_max_	0.394, 0.746
No. of measured, independent and observed [*I* > 2σ(*I*)] reflections	19963, 1962, 1830
*R*_int_	0.032
(sin θ/λ)_max_ (Å^−1^)	0.617

Refinement	
*R*[*F* ^2^ > 2σ(*F* ^2^)], *wR*(*F* ^2^), *S*	0.032, 0.077, 1.14
No. of reflections	1962
No. of parameters	144
H-atom treatment	H-atom parameters constrained
Δρ_max_, Δρ_min_ (e Å^−3^)	0.35, −0.35

Computer programs: *APEX2* and *SAINT* (Bruker, 2007 ▸), *SHELXT2014* (Sheldrick, 2015*a* ▸), *SHELXL2016* (Sheldrick, 2015*b* ▸), *ORTEP-3 for Windows* and *WinGX* (Farrugia, 2012 ▸) and *PLATON* (Spek, 2009 ▸).

## supplementary crystallographic information

### Crystal data

**Table:**

[Co(C_6_H_6_N_2_O)_2_(H_2_O)_4_](C_4_H_2_O_4_)	*F*(000) = 506
*M**_r_* = 489.30	*D*_x_ = 1.604 Mg m^−^^3^
Monoclinic, *P*2_1_/*c*	Mo *K*α radiation, λ = 0.71073 Å
*a* = 9.6914 (10) Å	Cell parameters from 9553 reflections
*b* = 10.0106 (11) Å	θ = 3.1–28.3°
*c* = 11.3811 (12) Å	µ = 0.91 mm^−^^1^
β = 113.416 (3)°	*T* = 296 K
*V* = 1013.22 (19) Å^3^	Block, orange
*Z* = 2	0.25 × 0.19 × 0.16 mm

### Data collection

**Table:**

Bruker APEXII CCD diffractometer	1830 reflections with *I* > 2σ(*I*)
φ and ω scans	*R*_int_ = 0.032
Absorption correction: analytical (X-RED32; Stoe & Cie, 2002)	θ_max_ = 26.0°, θ_min_ = 3.1°
*T*_min_ = 0.394, *T*_max_ = 0.746	*h* = −11→11
19963 measured reflections	*k* = −12→12
1962 independent reflections	*l* = −14→13

### Refinement

**Table:**

Refinement on *F*^2^	0 restraints
Least-squares matrix: full	Hydrogen site location: mixed
*R*[*F*^2^ > 2σ(*F*^2^)] = 0.032	H-atom parameters constrained
*wR*(*F*^2^) = 0.077	*w* = 1/[σ^2^(*F*_o_^2^) + (0.0211*P*)^2^ + 1.1735*P*] where *P* = (*F*_o_^2^ + 2*F*_c_^2^)/3
*S* = 1.14	(Δ/σ)_max_ < 0.001
1962 reflections	Δρ_max_ = 0.35 e Å^−^^3^
144 parameters	Δρ_min_ = −0.35 e Å^−^^3^

### Special details

**Table:**

Geometry. All esds (except the esd in the dihedral angle between two l.s. planes) are estimated using the full covariance matrix. The cell esds are taken into account individually in the estimation of esds in distances, angles and torsion angles; correlations between esds in cell parameters are only used when they are defined by crystal symmetry. An approximate (isotropic) treatment of cell esds is used for estimating esds involving l.s. planes.

### Fractional atomic coordinates and isotropic or equivalent isotropic displacement parameters (Å^2^)

**Table:**

	*x*	*y*	*z*	*U*_iso_*/*U*_eq_	
Co1	0.500000	0.500000	0.500000	0.01658 (12)	
O3	0.60307 (15)	0.65750 (13)	0.62150 (13)	0.0237 (3)	
H3A	0.696404	0.660264	0.635017	0.035*	
H3B	0.594575	0.646860	0.692916	0.035*	
O2	0.39661 (15)	0.64187 (13)	0.35319 (13)	0.0240 (3)	
H2A	0.368923	0.604165	0.279836	0.036*	
H2B	0.458953	0.704445	0.358200	0.036*	
O4	0.55105 (17)	0.63859 (14)	0.83392 (14)	0.0283 (3)	
O1	−0.09235 (17)	0.70328 (16)	0.68073 (17)	0.0365 (4)	
O5	0.72605 (18)	0.48025 (16)	0.88934 (16)	0.0356 (4)	
N1	0.31337 (18)	0.52596 (16)	0.55744 (16)	0.0208 (3)	
C7	0.6189 (2)	0.54056 (19)	0.90123 (18)	0.0221 (4)	
C3	0.1007 (2)	0.5713 (2)	0.66124 (18)	0.0216 (4)	
C8	0.5656 (2)	0.48648 (19)	0.99864 (19)	0.0239 (4)	
H8	0.629871	0.429951	1.061428	0.029*	
N2	−0.0347 (3)	0.5112 (2)	0.7922 (2)	0.0491 (6)	
H2C	−0.102155	0.523521	0.822413	0.059*	
H2D	0.020826	0.440936	0.812861	0.059*	
C4	0.1453 (2)	0.6727 (2)	0.6015 (2)	0.0268 (4)	
H4	0.103978	0.757665	0.594549	0.032*	
C2	0.1627 (2)	0.4464 (2)	0.6655 (2)	0.0297 (5)	
H2	0.134441	0.375248	0.703564	0.036*	
C6	−0.0163 (2)	0.6003 (2)	0.7141 (2)	0.0279 (4)	
C5	0.2516 (2)	0.64642 (19)	0.5523 (2)	0.0263 (4)	
H5	0.281757	0.715892	0.513627	0.032*	
C1	0.2671 (2)	0.4283 (2)	0.6126 (2)	0.0275 (4)	
H1	0.307396	0.343382	0.615691	0.033*	

### Atomic displacement parameters (Å^2^)

**Table:**

	*U*^11^	*U*^22^	*U*^33^	*U*^12^	*U*^13^	*U*^23^
Co1	0.01724 (19)	0.01624 (19)	0.0214 (2)	−0.00028 (12)	0.01310 (14)	0.00056 (13)
O3	0.0239 (7)	0.0251 (7)	0.0276 (7)	−0.0036 (6)	0.0162 (6)	−0.0033 (6)
O2	0.0259 (7)	0.0220 (7)	0.0258 (7)	−0.0015 (5)	0.0121 (6)	0.0031 (5)
O4	0.0421 (8)	0.0219 (7)	0.0319 (7)	0.0059 (6)	0.0264 (7)	0.0046 (6)
O1	0.0332 (8)	0.0303 (8)	0.0602 (11)	−0.0001 (7)	0.0334 (8)	−0.0075 (7)
O5	0.0354 (8)	0.0430 (9)	0.0409 (9)	0.0129 (7)	0.0284 (7)	0.0091 (7)
N1	0.0207 (8)	0.0199 (8)	0.0274 (8)	−0.0003 (6)	0.0154 (7)	−0.0003 (6)
C7	0.0262 (9)	0.0217 (9)	0.0234 (9)	−0.0018 (8)	0.0151 (8)	−0.0023 (8)
C3	0.0178 (9)	0.0268 (10)	0.0247 (9)	−0.0017 (7)	0.0134 (7)	−0.0023 (8)
C8	0.0282 (10)	0.0240 (10)	0.0243 (10)	0.0033 (8)	0.0155 (8)	0.0037 (7)
N2	0.0411 (12)	0.0675 (15)	0.0591 (14)	0.0163 (11)	0.0416 (11)	0.0225 (11)
C4	0.0285 (10)	0.0182 (9)	0.0425 (12)	0.0012 (8)	0.0234 (9)	−0.0016 (8)
C2	0.0303 (10)	0.0270 (10)	0.0428 (12)	0.0024 (9)	0.0261 (10)	0.0110 (9)
C6	0.0208 (9)	0.0359 (12)	0.0330 (11)	−0.0056 (9)	0.0170 (8)	−0.0089 (9)
C5	0.0300 (10)	0.0191 (9)	0.0397 (11)	−0.0005 (8)	0.0245 (9)	0.0032 (8)
C1	0.0274 (10)	0.0204 (10)	0.0432 (12)	0.0039 (8)	0.0229 (9)	0.0058 (8)

### Geometric parameters (Å, º)

**Table:**

Co1—O3^i^	2.0731 (13)	C7—C8	1.498 (3)
Co1—O3	2.0731 (13)	C3—C2	1.380 (3)
Co1—O2^i^	2.1171 (13)	C3—C4	1.383 (3)
Co1—O2	2.1171 (13)	C3—C6	1.509 (3)
Co1—N1	2.1694 (16)	C8—C8^ii^	1.313 (4)
Co1—N1^i^	2.1694 (16)	C8—H8	0.9300
O3—H3A	0.8556	N2—C6	1.320 (3)
O3—H3B	0.8555	N2—H2C	0.8600
O2—H2A	0.8564	N2—H2D	0.8600
O2—H2B	0.8564	C4—C5	1.380 (3)
O4—C7	1.258 (2)	C4—H4	0.9300
O1—C6	1.236 (3)	C2—C1	1.379 (3)
O5—C7	1.254 (2)	C2—H2	0.9300
N1—C1	1.332 (3)	C5—H5	0.9300
N1—C5	1.337 (2)	C1—H1	0.9300
			
O3^i^—Co1—O3	180.0	O4—C7—C8	118.86 (17)
O3^i^—Co1—O2^i^	88.15 (6)	C2—C3—C4	117.75 (17)
O3—Co1—O2^i^	91.85 (6)	C2—C3—C6	123.14 (18)
O3^i^—Co1—O2	91.85 (6)	C4—C3—C6	119.07 (18)
O3—Co1—O2	88.15 (6)	C8^ii^—C8—C7	124.4 (2)
O2^i^—Co1—O2	180.0	C8^ii^—C8—H8	117.8
O3^i^—Co1—N1	93.08 (6)	C7—C8—H8	117.8
O3—Co1—N1	86.92 (6)	C6—N2—H2C	120.0
O2^i^—Co1—N1	91.85 (6)	C6—N2—H2D	120.0
O2—Co1—N1	88.15 (6)	H2C—N2—H2D	120.0
O3^i^—Co1—N1^i^	86.92 (6)	C5—C4—C3	119.29 (18)
O3—Co1—N1^i^	93.08 (6)	C5—C4—H4	120.4
O2^i^—Co1—N1^i^	88.14 (6)	C3—C4—H4	120.4
O2—Co1—N1^i^	91.85 (6)	C1—C2—C3	119.26 (18)
N1—Co1—N1^i^	180.0	C1—C2—H2	120.4
Co1—O3—H3A	109.8	C3—C2—H2	120.4
Co1—O3—H3B	109.6	O1—C6—N2	123.2 (2)
H3A—O3—H3B	109.1	O1—C6—C3	119.28 (19)
Co1—O2—H2A	109.9	N2—C6—C3	117.5 (2)
Co1—O2—H2B	109.8	N1—C5—C4	123.21 (18)
H2A—O2—H2B	109.1	N1—C5—H5	118.4
C1—N1—C5	117.00 (16)	C4—C5—H5	118.4
C1—N1—Co1	122.07 (13)	N1—C1—C2	123.47 (19)
C5—N1—Co1	120.48 (13)	N1—C1—H1	118.3
O5—C7—O4	124.37 (18)	C2—C1—H1	118.3
O5—C7—C8	116.71 (18)		
			
O5—C7—C8—C8^ii^	−161.6 (3)	C2—C3—C6—N2	15.6 (3)
O4—C7—C8—C8^ii^	15.7 (4)	C4—C3—C6—N2	−166.7 (2)
C2—C3—C4—C5	−1.8 (3)	C1—N1—C5—C4	0.4 (3)
C6—C3—C4—C5	−179.59 (19)	Co1—N1—C5—C4	−172.01 (17)
C4—C3—C2—C1	1.1 (3)	C3—C4—C5—N1	1.1 (3)
C6—C3—C2—C1	178.8 (2)	C5—N1—C1—C2	−1.2 (3)
C2—C3—C6—O1	−162.7 (2)	Co1—N1—C1—C2	171.10 (17)
C4—C3—C6—O1	14.9 (3)	C3—C2—C1—N1	0.5 (4)

Symmetry codes: (i) −*x*+1, −*y*+1, −*z*+1; (ii) −*x*+1, −*y*+1, −*z*+2.

### Hydrogen-bond geometry (Å, º)

**Table:**

*D*—H···*A*	*D*—H	H···*A*	*D*···*A*	*D*—H···*A*
O2—H2*A*···O5^i^	0.86	1.96	2.814 (2)	171
O2—H2*B*···O4^iii^	0.86	1.88	2.7165 (19)	165
O3—H3*A*···O1^iv^	0.86	1.95	2.792 (2)	168
O3—H3*B*···O4	0.86	1.82	2.6652 (19)	172
N2—H2*C*···O5^v^	0.86	2.13	2.955 (2)	160
N2—H2*D*···O1^vi^	0.86	2.47	3.288 (3)	159
C1—H1···O4^vii^	0.93	2.41	3.322 (2)	168
C2—H2···O1^vi^	0.93	2.30	3.225 (3)	173

Symmetry codes: (i) −*x*+1, −*y*+1, −*z*+1; (iii) *x*, −*y*+3/2, *z*−1/2; (iv) *x*+1, *y*, *z*; (v) *x*−1, *y*, *z*; (vi) −*x*, *y*−1/2, −*z*+3/2; (vii) −*x*+1, *y*−1/2, −*z*+3/2.
